# Influence of Immediate Dentin Sealing on the Shear Bond Strength of Pressed Ceramic Luted to Dentin with Self-Etch Resin Cement

**DOI:** 10.1155/2012/310702

**Published:** 2012-01-12

**Authors:** Robert Dalby, Ayman Ellakwa, Brian Millar, F. Elizabeth Martin

**Affiliations:** ^1^Faculty of Dentistry, The University of Sydney, NSW 2006, Australia; ^2^Restorative Dentistry, King's College London Dental Institute, London SE1 9RT, UK

## Abstract

*Objectives*. To examine the effect of immediate dentin sealing (IDS), with dentin bonding agents (DBAs) applied to freshly cut dentin, on the shear bond strength of etched pressed ceramic luted to dentin with RelyX Unicem (RXU) cement. *Method*. Eighty extracted noncarious third molars were ground flat to expose the occlusal dentin surfaces. The teeth were randomly allocated to five groups (A to E) of sixteen teeth each. Groups A to D were allocated a dentin bonding agent (Optibond FL, One Coat Bond, Single Bond, or Go!) that was applied to the dentin surface to mimic the clinical procedure of IDS. These specimen groups then had etched glass ceramic discs (Authentic) luted to the sealed dentin surface using RXU. Group E (control) had etched glass ceramic discs luted to the dentin surface (without a dentin bonding agent) using RXU following the manufacturer's instructions. All specimens were stored for one week in distilled water at room temperature and then shear stressed at a constant cross-head speed of 1 mm per minute until failure. Statistical analysis was performed by ANOVA followed by post hoc Tukey HSD method (*P* < 0.05) applied for multiple paired comparisons. *Results*. The shear bond strength results for group A to E ranged from 6.94 ± 1.53 to 10.03 ± 3.50 MPa. One-way ANOVA demonstrated a difference (*P* < 0.05) between the groups tested and the Tukey HSD demonstrated a significant (*P* < 0.05) difference between the shear bond strength (SBS) of Optibond FL (Group A) and Go! (Group D). There was no statistical difference (*P* > 0.05) in the SBS between the test groups (A–D) or the control (group E). *Conclusion*. IDS using the dentin bonding agents tested does not statistically (*P* > 0.05) affect the shear bond strength of etched pressed ceramic luted to dentin with RXU when compared to the control.

## 1. Introduction

The preparation of teeth for indirect bonded restorations involves the cutting of dentin and hence the exposure of dentinal tubules [1]. This, in turn, may result in pulpal injury or produce sensitivity [2]. More conservative approaches to restorative dentistry have been made possible by the advent of adhesive technology which also enables sealing of these exposed dentin tubules [3]. It is possible to seal these freshly cut dentin surfaces with a dentin bonding agent immediately after tooth preparation, before impression taking. Most studies on the bond strength of dentin bonding agents use freshly prepared dentin. In daily practice, teeth require provisional restorations to protect the dentin and provide for the patient's functional and aesthetic needs when providing indirect restorations. However, dentin contaminated with provisional cement can reduce the potential for dentin bonding [4].

Magne [5] has described a procedure called immediate dentin sealing (IDS) where a DBA, Optibond FL, is applied immediately after tooth preparation. This is in contrast to the common practice of delayed dentin sealing (DDS) where dentin bonding is carried out just prior to cementation of the definitive restoration. The claimed benefits include minimizing pulp irritation and less need for anesthesia on removal of the provisional crown, as well as an increase in bond strength. This latter finding following final cementation is in agreement with the work of Cherkasski and Wilson [6].

Dental bonding allows the use of resin-based luting cements in conjunction with dentin bonding agents. These resin cements can be either self-curing or photoactivated and commonly are both. There have been recent innovations in self-etching cements in an ongoing attempt to simplify the procedural steps with adhesives. Resin cements have been shown to bond consistently to prosthodontic materials that have received micromechanical surface treatments [7].

Resin cements' adhesion to tooth structure may be enhanced by the use of conventional DBAs, used with a self-etching primer (self-etch) or be self-adhesive. RelyX Unicem (RXU), the self- adhesive cement used in the current study, has been demonstrated to have a shear bond strength comparable to conventional resin cements used with DBAs and self-etch resins [8].

Unlike the DBAs that utilize an etch and rinse system, self-adhesive cements, such as RXU, do not dissolve the smear layer and do not form a hybrid layer or form resin tags in the tubules [9].

However, unlike other self-etching cements, RXU demonstrates a rapid rise to neutral pH on setting [10].

RXU has been shown to have enhanced bond strength to acid-etched enamel [11]. However, the manufacturer's instructions specifically recommend no dentin pretreatment apart from cleaning with pumice and water. Other studies have also shown that the bond strength of RXU to dentin is decreased if the dentin is acid etched prior to application [7].

To date there has been minimal investigation of the effect of pretreatment of exposed dentin with DBAs prior to cementation with RXU.

Given that the application of DBAs is a recognized procedure for dentin sealing, the aim of this study was to investigate the effect of dentin sealing with four commonly used DBAs on the shear bond strength of RXU cement compared to a control (without bonding agent). The null hypothesis was that the immediate application of precured dentin bonding agent following tooth preparation does not significantly alter the shear bond strength of etched glass ceramic luted to dentin with RelyX Unicem.

## 2. Materials and Methods

The bonding agents, etched glass ceramic and RXU, with batch numbers and composition are listed in Table 1. Eighty noncarious, freshly extracted third molar teeth were collected from consenting patients. The teeth were manually cleaned and then placed in 5% sodium hypochlorite for fifteen minutes. These were then washed and stored in distilled water under refrigeration. Teeth were not stored for longer than three months. Preparation of the tooth surfaces involved initially removing the occlusal surface enamel using a model trimmer under water irrigation. The presence of residual enamel was visually evaluated under 5x magnification and removal ensured. The teeth were then mounted in plastic rings of 26 mm internal diameter and 25 mm height and embedded in epoxy resin (West System 105 Epoxy, Adhesive Technologies Ltd, Henderson, Auckland). The specimens were then sequentially hand sanded with a 600-grit silicon carbide paper under water to create a smooth dentin surface and a smear layer.

The glass ceramic “Authentic” (Ceramay, Stuttgart, Germany) is a heat-pressed leucite reinforced glass ceramic porcelain. This porcelain has a Vicker's hardness of 620 and an average particle size of 4 *μ*m as opposed to other veneer porcelains with a Vicker's hardness of 7–900 (and is hence less damaging to the opposing teeth). It has flexural modulus of 90 GPa as opposed to dentin at 18–20 GPa [12].

The “Authentic” ceramic was cast in 5 mm diameter cylinders that were then hand cut with a diamond disc into 3 mm sections. These ceramic discs were etched following the manufacturer's recommendations with hydrofluoric acid for 14 minutes (HF Etchant: American Dental Supplies Inc.). After etching the discs were washed and ultrasonically cleaned in distilled water for 15 minutes to remove any surface contaminants resulting from the etching process.

Cellophane adhesive tape of 40 *μ*m thickness with a standardized 2.4 mm diameter circular perforation was then placed over the exposed dentin surface of each sectioned tooth. This provided a standardized film thickness and area of bonding for the cement.

Ideally the film thickness of luting cements should be minimal to reduce exposure of the cement to the oral fluids, minimize polymerization contraction, and allow for good fit of the restoration. Optimal film thickness is a point of some conjecture. Previously a film thickness of 25 *μ*m was considered appropriate when frictional cements such as zinc phosphate were the material lute of choice. With adhesive resin cements a film thickness of up to 75 *μ*m may now be considered acceptable. The thickness of the cellophane is therefore a reasonable compromise for the clinical setting [13].

The teeth were divided randomly into five groups of sixteen teeth coded A to E (Table 3). The dentin surfaces of all teeth were cleaned with a slurry of pumice and water. For groups A to D, one of the four test bonding agents (Optibond FL, One Coat Bond, Single Bond, Go!) was applied to the dentin surface, respectively, to mimic the clinical procedure of IDS following the manufacturer's instructions, as described in Table 2. The only variation was the use of high-volume suction to dry the dentin prior to the application of Optibond FL (group A) to emulate the IDS procedure as proposed by Magne [5]. Group E was prepared for RXU following the manufacturer's instructions (Table 2) and was assigned as the control.

A single operator applied the DBAs to the exposed dentin surface of each specimen and cemented the ceramic discs with RXU, using premeasured capsules, to the dentin surfaces under firm finger pressure. The cement was immediately photopolymerized for 20 seconds in “ramp mode” from four directions parallel to the cement interface and finally through the ceramic for a further 20 seconds with a high-powered LED curing light (Radii Plus, SDI, USA). The “ramp mode” is designed to allow a gradual increase in intensity for the first 5 seconds to a peak intensity of 1500 mW/cm^2^ for the remaining 15 seconds and purports to minimize polymerization stress [14].

The test specimens were immediately placed in distilled water and stored at room temperature for one week. A number of specimens failed during water storage and the remaining numbers of specimens were shear tested for each group (Table 3).

To conduct the shear bond strength (SBS) test each of the remaining specimens was individually mounted in a jig and tested using a Shimadzu AG-50 kNE universal testing machine utilizing a 1 kN load cell with a range set at 0–100 newtons. The test speed used was 1 mm/min. The shear bond force was recorded in newtons and converted to megapascals (MPa) to represent bond strength. Specimens that failed before actual testing were attributed a bond strength of 0 MPa. The data of the SBS were subjected to analysis by one-way ANOVA. The Tukey HSD test was used for multiple paired comparisons (*α* = 0.05).

Following the testing the ceramic and tooth surface of all specimens was examined under 100x light magnification (Leica DC-100, Meyer Instruments, Houston, TX, USA) and photographed in order to determine the mode of failure. These were then categorized as one of four modes of failure:

mode failure 1: adhesive failure at the ceramic-cement interface,
mode failure 2: adhesive failure at the bonding surface-cement interface,
mode failure 3: cohesive failure within the cement, or
mode failure 4: mixed failure (any combination of the first three modes).

A further 10 specimens were prepared for SEM examination of the dentin-cement-ceramic junction. This involved preparing two specimens each of the test bonding systems and two of the control. These specimens were then set horizontally in larger perspex rings using epoxy resin. The specimens were then sectioned with a diamond blade under water cooling and the surface further sanded with silicon-carbide sand paper to 1 micron grit for SEM examination.

## 3. Results

The shear bond strength (SBS) results are presented in Table 3. The ANOVA test demonstrated a difference between the groups tested that was further compared with the Tukey HSD test to show a significant (*P* < 0.05) difference between groups A (Optibond FL) and D (Go!). There was no significant (*P* > 0.05) difference between groups A to D and the control (group E) and there was also no significant (*P* > 0.05) difference between groups B, C, and D.

**Figure 1:**
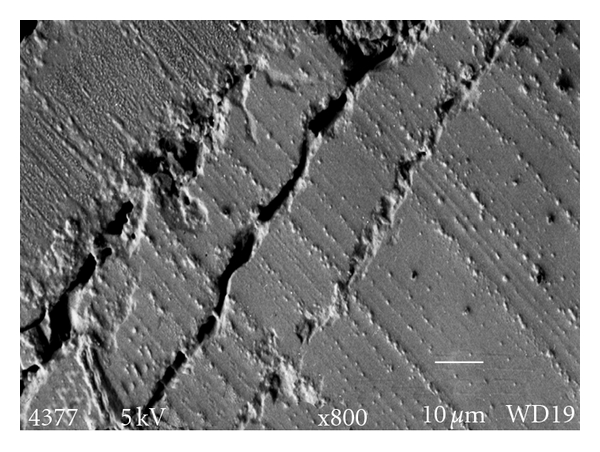
SEM demonstrating the fracture interface surface between the ceramic surface and the luting cement (mode 1).

**Figure 2:**
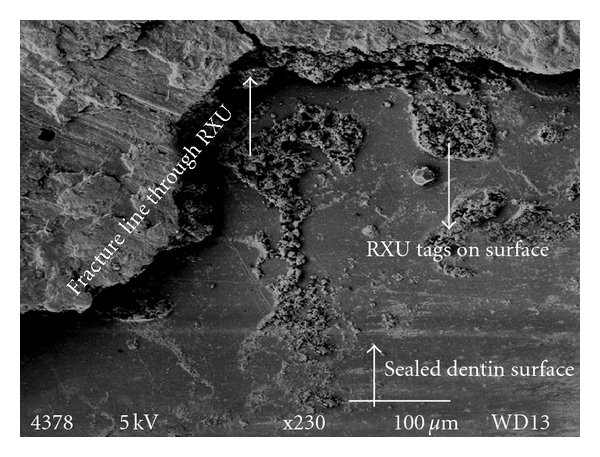
Shows a mixed fracture interface surface (mode 4). The dentinal surface is sealed and cracks propagate through the luting cement (arrows).

The failure modes were predominantly identified as mode 1 (failure of the adhesive at the ceramic cement interface) or mode 4 (mixed failure). Examples of the modes of failure are shown in Figures 1 and 2.  Figure 1 shows the fracture interface surface between the ceramic and luting cement (mode 1) and Figure 2 shows a mixed fracture interface surface (mode 4). In this image the dentinal surface appears sealed and cracks propagate through the luting cement (arrow).

**Figure 3:**
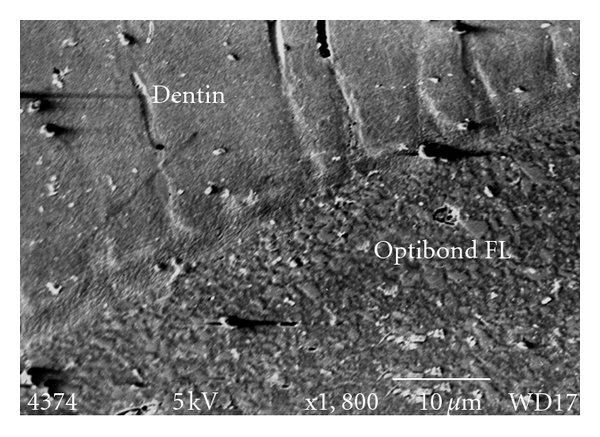
Optibond FL tooth interface demonstrating the hybrid layer.

**Figure 4:**
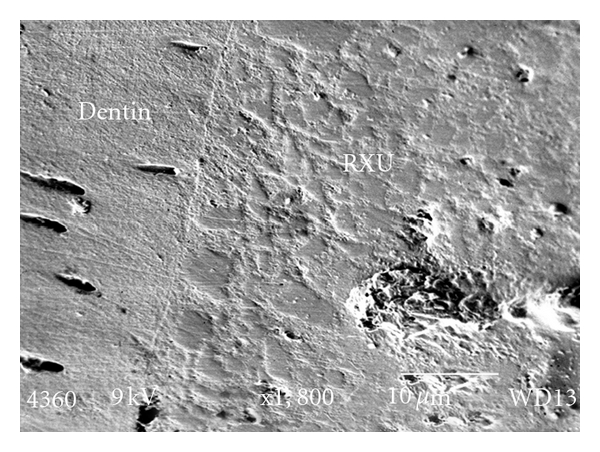
The dentin RXU interface showing no hybrid layer formation.

 Optibond FL (Group A) demonstrated the highest mean SBS. This result is significantly different (*P* < 0.05) from the SBS of Go! (Group D), but not to the control (RXU/group E).  Figure 3 shows a cross-section through the Optibond FL/tooth interface with formation of hybrid layer.  Figure 4 shows the dentin -RXU interface with little or no hybrid layer formation.

As represented in Table 3 there is a predominance of mixed interface failure (51.85%) followed by failure at the ceramic-cement interface (37.04%). Half the RXU specimens (Group E) demonstrated mixed mode failure with three failures at the ceramic-cement interface and four at the bonding surface cement interface. There were two failures during water storage that were both ceramic-cement failures.

**Figure 5:**
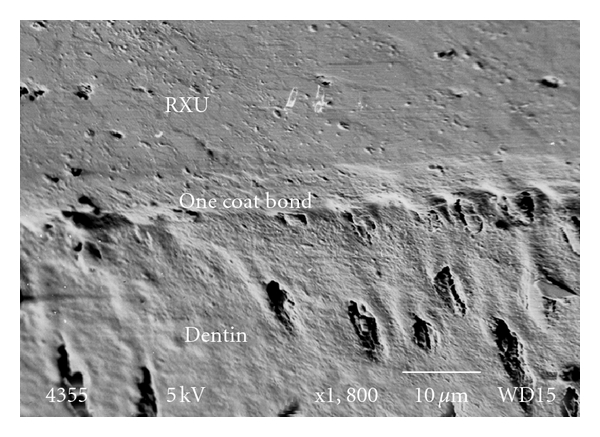
The interface of dentin/One Coat Bond DBA/RXU.

The One Coat Bond specimens (Group B) mainly exhibited failure at the ceramic-cement interface with one failure occurring during water storage.  Figure 5 shows a cross-section of the interface illustrating hybrid layer formation between the OCB and the overlying RXU.

The Single Bond group (Group C) also demonstrated predominantly adhesive failure at the ceramic-cement interface. There were two failures during water storage.

Go! specimens (Group D) showed a different pattern of mostly mixed failures with minimal resin remaining on the ceramic surface when viewed under 100x magnification. This group had the largest number of bond failures occurring during water storage and all were ceramic-cement failures. There was no correlation found between fracture mode and the SBS for individual specimens.

## 4. Discussion

This study was designed to investigate the effect of immediate dentin sealing of freshly cut dentin on the shear bond strength of a commonly used self-etching resin cement to lute etched glass ceramic to treated dentin surfaces. The dentin surfaces were treated with different bonding agents, the glass ceramic discs luted in place and then the samples stored in distilled water (room temperature) for 7 days prior to testing. The null hypothesis was that the immediate application of a dentin bonding agent following tooth preparation does not significantly alter the shear bond strength of etched glass ceramic luted to dentin with RXU.

The results appear to support the null hypothesis with no significant difference (*P* > 0.05) being demonstrated between the mean shear bond strengths of the different specimen compared to the control specimens, where no bonding was carried out prior to luting with RXU (Group E).

It has been reported that the RXU exhibits a form of ionic bonding to dentin [15]. The authors of this study hypothesized that demineralizing the dentin surface with any form of acid treatment would remove surface mineral content of the dentin and hence a lower bond strength would be expected. This correlates with the results from previous studies showing that RXU has a lower bond strength to acid-etched dentin compared with untreated dentin [14]. Acid surface treatments would potentially leave a layer of mineral depleted collagen fiber matting in contact with RXU, which would reduce ionic exchange.

Using DBAs with RXU will result in a hybrid layer being interposed between the RXU and the mineralized dentin possibly acting as a barrier to inhibiting the formation of ionic bonds. This alternately is countered by the resin component of the RXU bonding to the resin component of the hybrid layer, hence the recorded bond strengths in this study. The use of surface treatments and application of DBAs would logically revert the luting process to that of using a non-self-etching resin with DBAs.

Along these lines the use of IDS would be expected to give suitable bond strengths with RXU as demonstrated in this study. The exception in the current study was with group D (Go!). It could be hypothesized that the poor results found with Go! (Group D) may be due to a possible material incompatibility between the single-bottle self-etch DBA and dual-cured composites resulting in a reduced chemical interaction between the resin hybrid layer and the RXU cement [15].

There are various methods used to test the bond strength of cements to substrate and shear testing was used in this study. Fowler et al. [16] and Oilo & Austrheim [17] found that shear and tensile bond testing of adhesives produced comparable results. Alternately, Kitasako et al. [18] demonstrated differences in adhesive strengths of resin cements bonded to dentin when comparing shear and tensile testing. All three studies concluded that either test is valid; however, each agreed that the failure modes vary considerably with the differing test modes. 

The shear bond strengths to dentin for RXU in this study were lower than those achieved by other researchers; however other authors have cited difficulties in comparing shear test results from different machines [19].

The finding that there was no statistically significant reduction (*P* > 0.05) in bond strength with the dentin sealing procedures prior to the application of the RXU is an interesting result. This is contrary to the manufacturer's instructions for the use of RXU that recommends no pretreatment, other than cleaning the tooth surface with pumice slurry.

The results indicate that use of the IDS procedure as advocated by Magne [5] may have some positive effect on shear bond strengths to dentin after storage in water for one week. There was a statistically significant difference (*P* < 0.05) in SBS between using Optibond Fl (a 3-step etch rinse DBA/Group A) and using Go! (1-step self-etch DBA/Group D) for immediate dentin sealing. While bond strength data for the newer one-bottle systems are limited, the results of this study correlate with those of a recent study demonstrating that multistep bonding produced higher bond strengths to dentin [20].

 Results from the current study have shown that using a DBA with higher filler content (Group A) increases the bond strength; however it would be reasonable to assume that increasing the filler content may decrease the flexibility of the bonding agent and that an unfilled resin DBA would improve wettability and allow a more even stress distribution and thus give higher SBS results [21]. This would appear to be in contrast to the results demonstrated by the difference in mean SBSs between the highly filled DBA Optibond FL [22] (Group A) and the minimally filled Go! (Group D) [23] in the current study. The next highest mean SBS value was for Single Bond (Group C) which also has “nanofillers” added [24]. One Coat Bond (Group B) is also “nanofilled” to lower percentage [25]. The RXU itself has 70% filler content [26]. RXU does have a high filler content but does not need to penetrate dentin to form the “hybrid layer” [13].

The mode of failure in the current study was predominantly mixed failure/mode 4 (51.9%) followed by failure at the ceramic-cement interface/mode 1 (31.5%). There was only one cohesive failure/mode 3 recorded within the RXU (Group E) and only 16.7% of specimens failed at the cement dentin interface/mode 2. It might therefore be interpreted that the dentin bond strength would be stronger than the ceramic-cement bond. However this does not correlate with the finding of other researchers who found that shear bond strengths of RXU to etched glass ceramics approached 22 MPa for light-cured specimens [7]. The reason for this anomaly relates to specimen geometry and resultant stress distribution in the test materials. This is shown in Figure 1 where the fracture is seen within the ceramic and leaves the ceramic-cement interface intact. Other studies have found that conventional shear tests result in cohesive fractures within the dentin or the ceramic and contest that this is not representative of the true adhesive strength of the DBA to dentin; in addition varied SBS test results are noted which may be due to differing dentinal surface characteristics [17].

The DBA Go! (Group D) used in this study produced the lowest mean bond strength and interestingly a relatively large number of spontaneous failures during water storage. The one-step SE bonding agents have been previously reported to have lower bond strengths than multistep DBAs [27]. This may be a result of a particular technique sensitivity for the material or possibly an adverse interaction between this particular DBA and the RXU. Sanares et al. [28] have investigated such adverse interactions and found that the more acidic the self-etch DBA, the greater the decrease in bond strength to self-cured composites. These authors have postulated that residual acidic resin monomers from the adhesive inhibition layer of the self-etch DBA react with the binary peroxide-amine catalyst that is commonly employed in chemical-cured composite.

There may also be issues with the 1-step self-etching DBAs being too hydrophilic resulting in an incompatibility with chemical or dual-cure composite luting agents [29]. RXU is a dual-cure material. Chemically cured resins are commonly used as a restorative material in areas that are not easily accessed by light or as auto- or dual-curing resin cements for the luting of posts and restorations. It could be hypothesized that this incompatibility may be an issue for RXU in this study where it is setting as a chemically cured material in areas inaccessible to light. This chemical curing may occur where light curing cannot access the tooth surface that has been treated with a self-etch DBA. This may be responsible for the number of spontaneous specimen failures seen in our testing. If this is a real issue of material interaction, this would obviously be more relevant for areas inaccessible to light polymerization.

 Further research is needed to test the durability of the interface between the cement, glass ceramic, and tooth after longer periods of water storage.

## 5. Conclusion

Within the limits of this study the following can be concluded.

1. The application of the tested DBAs to dentin as an IDS procedure has no statistically detrimental effect (*P* > 0.05) on the SBS of RXU resin cement luting etched glass ceramic to dentin. Use of a DBA with RUX is in contrast to the manufacturer's instructions.
2. The IDS procedure using Optibond FL showed a statistically significant (*P* < 0.05) improvement in SBS when compared to the self-etch single-bottle adhesive Go! as used in this study.

## Figures and Tables

**Table 1:** Materials, manufacturers, and constituents.

Material	Manufacturer	Constituents
Optibond FL	Kerr Corporation. Orange, CA, USA. Lot number 3087960	Kerr Gel Etchant: 37.5% H_3_PO_4_; Primer: HEMA, GPDM, MMEP, water, ethanol, CQ, BHT; Adhesive: Bis-GMA, HEMA, GDMA, CQ, ODMAB, Filler (fumed SiO_2_, barium aluminoborosilicate, Na_2_SiF_6_), coupling factor A174 (approximately 48 wt % filled).

One Coat Bond	Coltene/Whaledent AG, Switzerland. Lot number 0159330	Bottle 1 = Primer: Acryloamidosulfonic acid, HEMA, glycerol mono- and dimethacrylate, polyalkenoate mehtacrylized. Bottle 2 = Adhesive: Hydroxyl mehtacrylate, UDMA, polyalkenoate methacrylized. (approximately 15 wt % filled).

Single Bond	3 M Espe. St. Paul, MN., USA. Lot number 20081211	BisGMA, HEMA, dimethacrylates, ethanol, water, photoinitiator system and a methacrylate functional copolymer of polyacrylic and poly (itaconic) acids. (approximately 10 wt % filled).

Go!	SDI, Brazil. Lot number 155740	Phosphoric acid ester monomer, Dimethacrylate monomer, Monomethacrylate monomer, Silicon dioxide filler Water, Acetone, Photoinitiators and Stabilizer, Sodium Fluoride (approximately 7 wt % filled).

RXU	3 M Espe. Seefeld, Germany. Lot number 347229	Multifunctional, phosphoric acid modified methacrylate monomers. (approximately 70 wt % filled).

“Authentic” Glass ceramic	Manufactured by Ceranay, Stuttgart, Germany. Lot number 1644554	Type II Class I. Leucite-reinforced Glass ceramic A4 Shade. Composition: 97–99.9% (SiO_2_, Al_2_O_3_, K_2_O, Na_2_O, CaO, B_2_O_3_, CeO_2_, TiO_2_, BaO) 0.1–3% (Pigments).

**Table 2:** Bonding systems and RXU application procedures.

Optibond FL 3-step Etch and Rinse	(1) Apply 37.5% phosphoric acid to dentin surface for 15 seconds. (2) Etchant rinsed with air and water spray for 20 seconds. Gentle air drying for 5 seconds. Care taken not to desiccate the dentin. (3) Apply OptiBond FL Prime (Bottle #1) to dentin surfaces with a light scrubbing motion for 15 seconds. Gently air dry with the high-volume suction for approximately 5 seconds. (4) Apply OptiBond FL Adhesive (Bottle #2) overexposed dentin uniformly creating a thin coating. (5) Light cure for 20 seconds. (6) Application of RelyX.

One Coat Bond 2-step Self-Etch	(1) Apply self-etching primer for 20 seconds applied with rubbing motion to dentin surface. (2) Air dry for 2 seconds. (3) Apply Bond for 20 seconds applied with rubbing motion to dentin surface. (4) Air dry for 2 seconds. (5) Light cure for 20 seconds. (6) Application of RelyX Unicem.

Single Bond 2-step Etch and Rinse	(1) Apply 37.5% phosphoric acid to dentin surface for 15 seconds. (2) Etchant rinsed with air and water for 20 seconds. (3) Dentin-blotted dry of excess water. (4) Apply 2 consecutive coats of single bond for 15 seconds each with gentle agitating. (5) Air dry for five seconds to evaporate solvent. (6) Light cure for 20 seconds. (7) Apply RelyX Unicem.

Go! 1-step Self-Etch	(1) Clean and blot dry tooth surface. (2) Apply to dentin for 20 seconds. (3) Air dry for 5 seconds. (4) Light cure for 10 seconds. (5) Apply RelyX Unicem.

RelyX Unicem (RXU)	(1) Clean tooth surface with pumice and water. (2) Air dry 2-3 seconds to remove pooled water. (3) Apply RelyX Unicem.

**Table 3:** Shear bond strength (MPa) ± SD of groups A to E/modes of failure.

Group code	Cement/bonding	No. of specimens failed	No. of specimens	Mean SBS ± SD	Mode of failure of tested specimens			
	agent	in water before testing	tested		1	2	3	4
A	Optibond FL	1	13	10.03 ± 3.50^a,1^	3	1	1	8
B	One Coat Bond	1	11	7.21 ± 2.83^1^	7	2	0	2
C	Single Bond	2	11	8.24 ± 3.35^1^	6	2	0	3
D	Go!	5	8	6.94 ± 1.53^b,1^	1	0	0	7
E (control)	RXU	2	11	7.17 ± 2.09^1^	1	2	1	7

Percentage of failures					37.04%	16.67%	1.85%	51.85%

Groups with the same superscript number in the same columns are not significantly different (*P* > 0.05).

Groups with a different superscript letter in the same column are significantly different (*P* < 0.05).

Mode failure 1: adhesive failure at the ceramic-cement interface, Mode failure 2: adhesive failure at the bonding surface-cement interface, Mode failure 3: cohesive failure within the cement or Mode failure 4: mixed failure (any combination of the first three modes).
